# Supplementary material for: Comparison between Intravoxel Incoherent Motion and Splenic Volumetry to Predict Hepatic Fibrosis Staging in Preoperative Patients
Source: Diagnostics (Basel). 2023 Oct 13;13(20):3200. doi: 10.3390/diagnostics13203200 (PMC10605488; doi:10.3390/diagnostics13203200)
Supplement: Supplementary file 1 [file diagnostics-13-03200-s001.zip › diagnostics-2610467-supplementary.pdf]

Table S1. **Comparison between the original data and our previously published paper [11].**

		Mean (Median)	Minimum	Maximum	25 percentile	75 percentile
**Patient data**						
Age	[11]	66.5 (69.0)	24	83	61	74
	CS	70.4 (72.0)	36	82	67	76
	P value	0.027				
BMI	[11]	23.0 (22.3)	17.6	31.5	20.6	25.2
	CS	23.6 (22.8)	17.5	33	20.9	26.1
	P value	0.356				
BSA	[11]	1.642 (1.637)	1.311	2.041	1.531	1.759
	CS	1.659 (1.626)	1.321	2.294	1.539	1.777
	P value	0.863				
Hct	[11]	40.8 (40.9)	33.3	49.7	38.2	43.4
	CS	39.7 (39.9)	29.5	50.7	36.8	42.7
	P value	0.178				
AST	[11]	35.4 (28.0)	15	153	22	38
	CS	35.4 (28.0)	12	153	21	42
	P value	0.853				
ALT	[11]	32.8 (28.5)	8	197	16.5	38.5
	CS	31.7 (21.0)	7	123	15	39
	P value	0.603				
Plt	[11]	18.8 (18.2)	6	47.8	13.6	22.9
	CS	16.0 (15.3)	4.3	31.7	11.6	20.1
	P value	0.024				
INR	[11]	1.03 (1.01)	0.87	1.26	0.98	1.08

	CS	1.01 (1.01)	0.91	1.29	0.97	1.04
	P value	0.167				
T bil	[11]	0.72 (0.64)	0.18	1.84	0.5	0.88
	CS	0.69 (0.64)	0.31	2.43	0.49	0.84
	P value	0.703				
Alb	[11]	4.4 (4.5)	3.5	5.3	4.1	4.8
	CS	4.2 (4.2)	3.3	5.2	4	4.4
	P value	0.001				
Cr	[11]	0.76 (0.74)	0.35	1.22	0.65	0.87
	CS	0.86 (0.79)	0.45	1.57	0.65	0.99
	P value	0.105				
ING_R15	[11]	11.703 (11.000)	0	71.1	5.7	14
	CS	13.624 (11.360)	3.5	77.7	7.7	16
	P value	0.116				
ALBI	[11]	-3.1 (-3.1)	-3.8	-2.3	-3.3	-2.9
	CS	-2.9 (-2.8)	-3.7	-2.1	-3.1	-2.7
	P value	<0.001				
MELD	[11]	2.3 (2.4)	-5	7.7	0.2	4.6
	CS	3.0 (2.5)	-4	10.4	0.7	5.3
	P value	0.288				
APRI	[11]	0.7 (0.5)	0.2	3	0.3	0.9
	CS	0.4 (0.4)	0.2	1.1	0.3	0.5
	P value	<0.001				
FIB_4	[11]	2.8 (2.2)	0.4	8.9	1.5	1.2

	CS	2.1 (2.1)	0.4	6.8	1.2	2.8
	P value	0.019				
SV	[11]	172.90 (143.97)	30.72	900.76	96.51	184.02
	CS	195.98 (171.65)	58	651.95	110	221
	P value	0.089				
SV/BSA	[11]	103.63 (86.37)	23.44	519.28	60.61	106.44
	CS	117.73 (100.04)	42.82	375.62	66.82	133.64
	P value	0.079				
			Male	Female	(+)	(-)
Sex	[11]		53	27		
	CS		49	18		
	P value	0.473				
HBV	[11]				22	58
	CS				15	52
	P value	0.568				
HCV	[11]				23	57
	CS				17	50
	P value	0.712				
Alcholic liver disease	[11]				8	72
	CS				9	58
	P value	0.608				
Others	[11]				3	77
	CS				4	63

	P value	0.702			
			5 point	6 point	7 point
Child Pugh score	[11]		78	2	0
	CS		62	4	1
	P value	0.306			

Notes: [11], previous study (n=80); CS, current study (n=67); BMI, body mass index; BSA, body surface area; Hct, hematocrit; AST, Aspartate transaminase; ALT, alanine aminotransferase; Plt, platelet; INR, International Normalized Ratio; T bil, Total billirubin; Alb, Albumin; Cr, Creatinine; ICG_R15, Indocyanine green retention 15min; ALBI, albumin-bilirubin; MELD, Mayo End-Stage Liver Disease; APRI, aspartate aminotransferase to platelet ratio index; SV, splenic volume; SV/BSA, ratio of splenic volume to body surface area; HBV, Hepatitis B Virus; HCV, Hepatitis C Virus.
